# Supplementary material for: Stable QTL for malate levels in ripe fruit and their transferability across Vitis species
Source: Hortic Res. 2022 Feb 28;9:uhac009. doi: 10.1093/hr/uhac009 (PMC8968676; doi:10.1093/hr/uhac009)
Supplement: Web_Material_uhac009 [file web_material_uhac009.zip › Supplementary tables and figures.pdf]

# Stable QTL for malate levels in ripe fruit and their transferability across *Vitis* species

Noam Reshef[1*], Avinash Karn[2], David C. Manns[3], Anna Katharine Mansfield[3], Lance Cadle-Davidson[4], Bruce Reisch[2], Gavin L. Sacks[1]

[1] Department of Food Science, Cornell University, Ithaca, NY 14853, USA
[2] Horticulture Section, School of Integrative Plant Science, Cornell University, Geneva, NY 14456, USA
[3] Department of Food Science, Cornell AgriTech, Geneva, NY 14456, USA
[4] USDA-ARS, Grape Genetics Research Unit, Geneva, NY 14456, USA
Correspondence to: nr444@cornell.edu

## Supplementary file 5

Supp. Table 1: Data transformations

Supp. Table 2 and 3: QTL identified for malate model parameters and fruit total soluble solid content (TSS)

Supp. Fig. 1: Effect plot of QTL found in the *V. rupestris* × 'Horizon' family

Supp. Fig. 2: Distribution of the change in fruit volume in 'Horizon' × Illinois 547-1 family

Supp. Fig. 3: Multi-year correlation of pre-veraison malate and TSS data

Supp. Fig. 4: Pathway enrichment analysis

Supp. Fig. 5: Reciprocal effect of malate QTL marker haplotypes between *V. rupestris* × 'Horizon' and 'Horizon' × Illinois 547-1

Supp. Fig. 6: Haplotype comparisons of the QTL peak markers among the parents and grandparents of *V. rupestris* × 'Horizon' and 'Horizon' × Illinois 547-1 families.

**Supplementary table 1.** Power transformation applied to ripe fruit malate data

| Year | Power transformation |
|---|---|
| **'Horizon'× Illinois 547-1** | |
| 2011 | 0.15 |
| 2012 | 0.4 |
| 2013 | 0.21 |
| 2018 | 0.27 |
| 2019 | None |
| BLUE | 0.34 |
| ***V. rupestris* B38 × 'Horizon'** | |
| 2012 | 0.22 |
| 2013 | 0.17 |
| BLUE | 0.05 |

**Supplementary table 2.** Summary of QTL associated with pre-veraison concentration ([Mal]green) and post-veraison degradation ([Mal]degradation) of malate during fruit development in 'Horizon' × Illinois 547-1 family

| Year | Phenotype | Power Transformation applied | N | Markers (Chr) Mb | LOD score[a] | LOD Interval[b] (Chr) Mb | %Variance QTL |
|---|---|---|---|---|---|---|---|
| 2019 | $[Mal]_{green}$ | ^1.97 | 133 | (7) 10.07 | 5.18** | (7) 5.3-18.84 | 16.43 |
| 2020 | $[Mal]_{green}$ | ^2.05 | 130 | NS | - | - | - |
| BLUE | $[Mal]_{green}$ | ^1.98 | 130 | (7) 15.37 | 5.85** | (7) 5.3-18.7 | 18.71 |
| 2019 | $[Mal]_{degradation}$ | None | 133 | (5) 9.1 | 3.96* | (5) 9.9-21.6 | 12.82 |

[a] LOD p values are represented by '*', '**', and '***' representing p<0.1, p<0.05, and p<0.01, respectively, based on 1,000 permutation tests. [b] 1.5-LOD drop-off confidence interval.

**Supplementary table 3.** Summary of QTL associated with TSS in 'Horizon' × Illinois 547-1 family

| Year | Power Transformation applied | N | Markers (Chr) Mb | LOD score[a] | LOD Interval[b] (Chr) Mb | %Variance QTL |
|---|---|---|---|---|---|---|
| 2011 | None | 73 | NS | - | - | - |
| 2012 | None | 147 | (17) 2.14 | 4.61* | (17) 0.29-2.5 | 13.5 |
| 2013 | None | 120 | (17) 4.13 | 4.82* | (17) 2.14-5.45 | 16.89 |
| 2018 | None | 116 | (6) 15.25 | 5.65** | (6) 6.02-18.52 | 20.11 |
| 2019 | None | 127 | NS | - | - | - |
| BLUE | None | 147 | (17) 5.36 | 5.27** | (17) 4.97-7.13 | 15.23 |

[a] LOD p values are represented by '*', '**', and '***' representing p<0.1, p<0.05, and p<0.01, respectively, based on 1,000 permutation tests. [b] 1.5-LOD drop-off confidence interval.

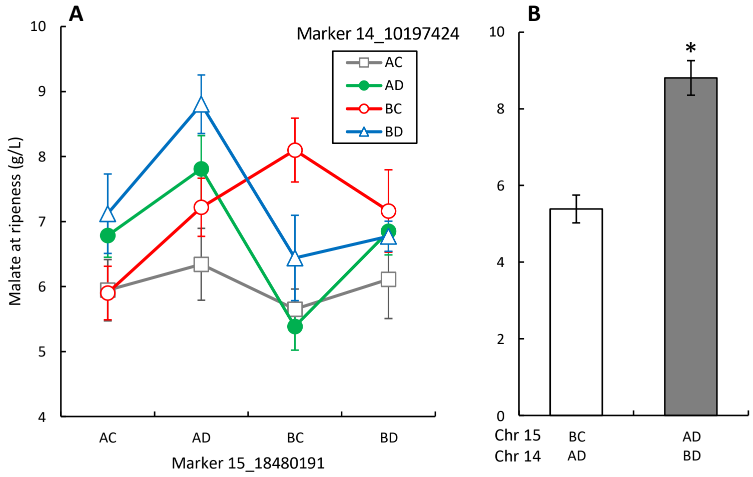


**Supplementary figure 1. QTL effect on grape berry malate levels at ripeness** in an interspecific grapevine (*Vitis*) mapping family, generated from a cross between *V. rupestris* and 'Horizon'. QTL analysis was performed using the best linear unbiased estimate (BLUE) calculated on two years of data (2012-2013). **A)** Effect of QTL marker haplotype combinations on fruit malate levels at ripeness, exhibiting the interaction between markers on chromosomes 14 and 15. **B)** Haplotype combinations of the two loci yielding highest and lowest mean malate concentrations at ripeness in the family, n=11 and 6, respectively. Error bars are standard errors. *Means significantly differ based on a Tukey HSD test, p value < 0.001.

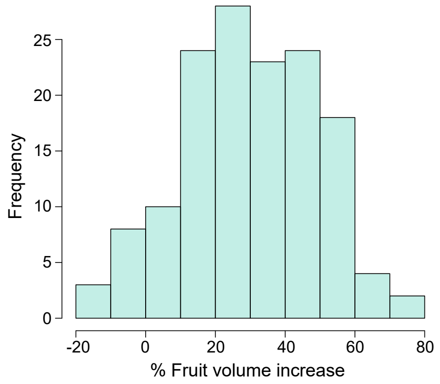


**Supplementary Figure 2. Distribution of the percent change in calculate fruit volume from pre-veraison to ripeness.** Values were measured in the 'Horizon'× Illinois 547-1 mapping family in 2019.

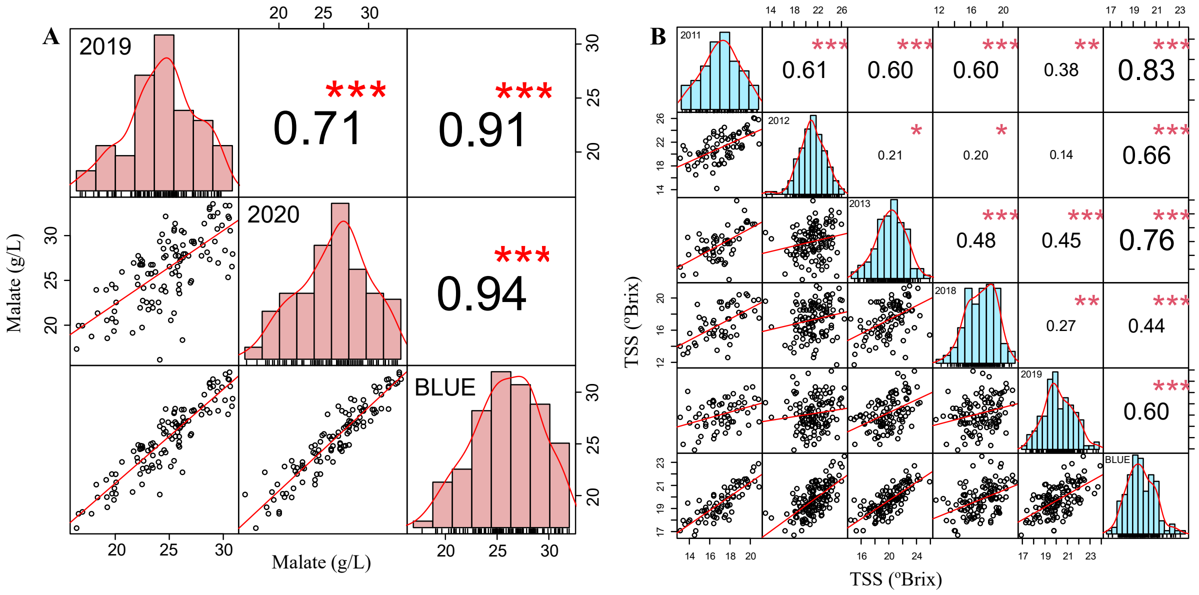


**Supplementary Figure 3. Multiyear correlation of malate at pre-veraison ($[Mal]_{green}$) (A) and fruit total soluble solids (TSS) at ripeness (B).** Pairwise correlations measured in each year and the calculated multi-year BLUE in the 'Horizon'× Illinois 547-1 mapping family. The upper-right diagonal panel shows pairwise Pearson's correlation coefficients with their corresponding p values represented by asterisks, where '*', '**', and '***' stands for $p<0.05$, 0.01, and 0.001, respectively. Bottom-left panel shows pairwise scatterplots, with red lines representing the best linear fit.

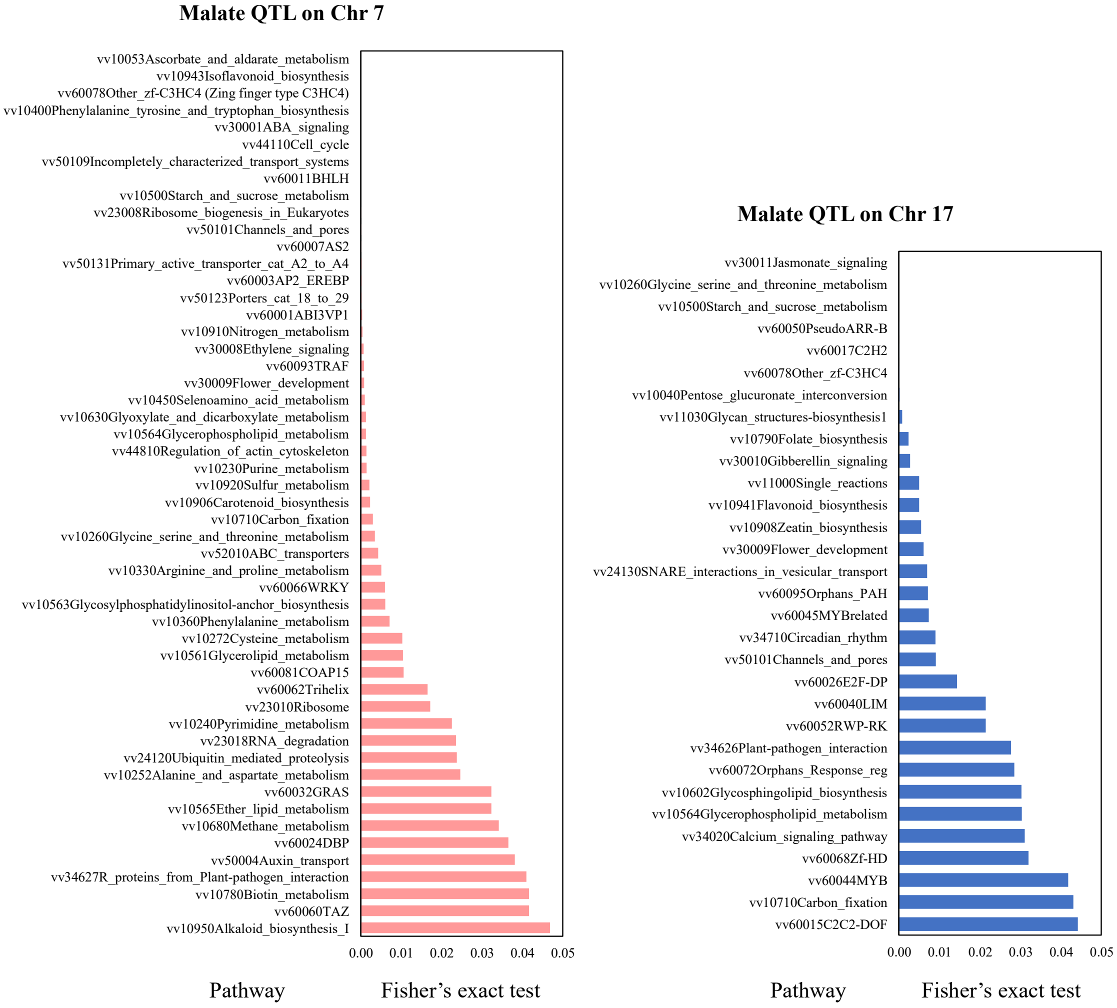


**Supplementary Figure 4. Pathway enrichment analysis** of genes located in the 1.5-LOD interval of the QTL for ripe fruit malate on chromosomes 7 and 17 ('Horizon'× Illinois 547-1 family).

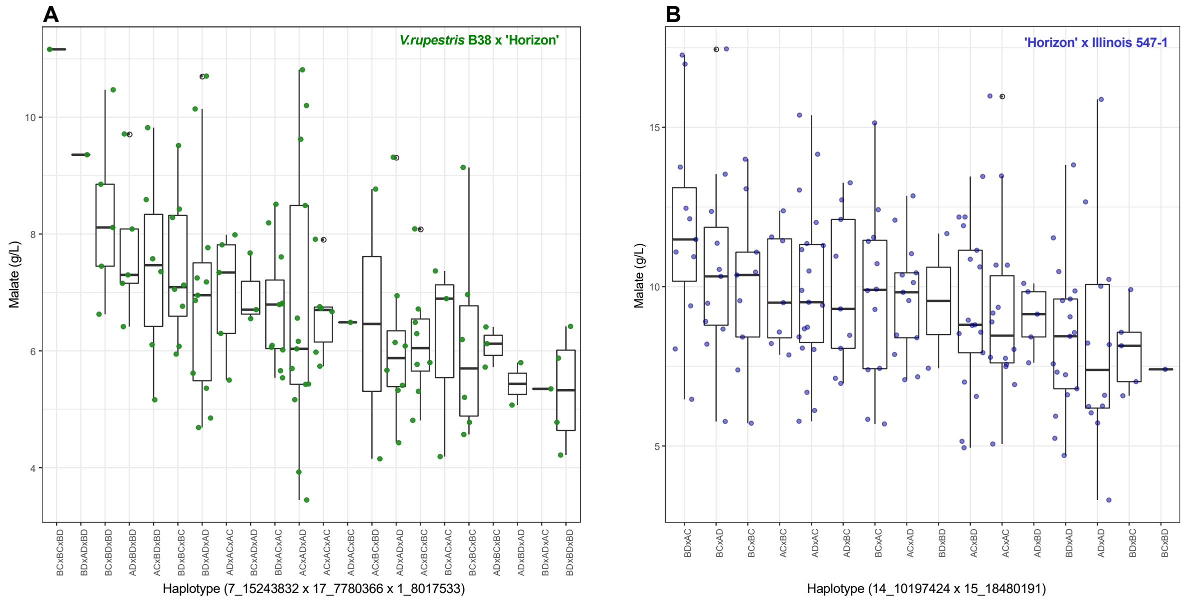


**Supplementary figure 5. Effect of malate QTL marker haplotypes found in one interspecific *Vitis* family on ripe fruit malate levels in a genetically related family. A)** Effect of haplotypes in QTL markers identified in ‘Horizon’ × Illinois 547-1 family on ripe fruit malate levels in *V. rupestris* B38 × ‘Horizon’ family. **(B)** Effect of haplotypes in QTL markers identified in *V. rupestris* B38 × ‘Horizon’ family on ripe fruit malate levels in ‘Horizon’ × Illinois 547-1 family. No statistically significant differences were found between groups based on ANOVA and Tukey HSD tests.

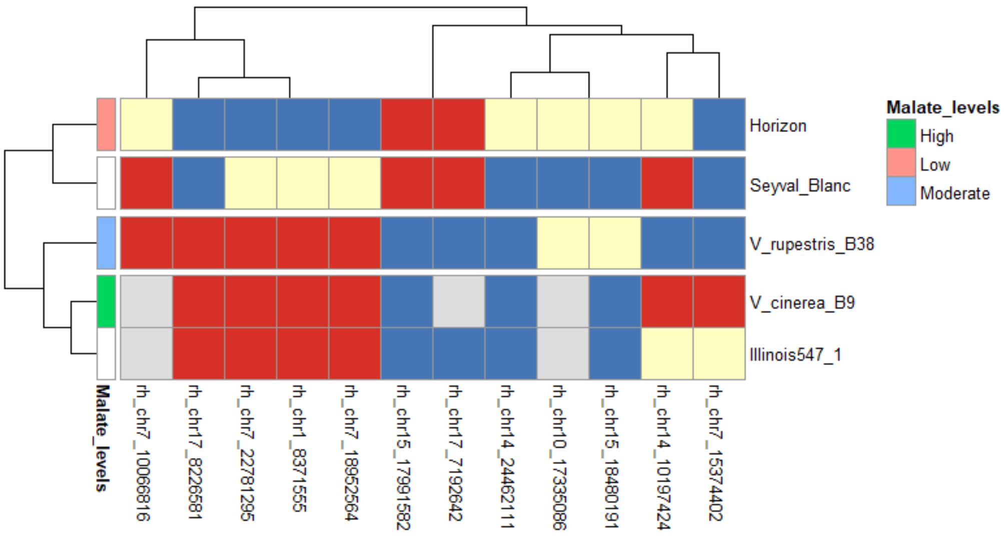


**Supplementary figure 6. Haplotype comparisons of the six QTL peak markers among the parents and grandparents of the *V. rupestris* B38 × ‘Horizon’ and ‘Horizon’ × Illinois 547-1 families.** *V. cinerea* B9 and Seyval Blanc are one of the parents of Illinois 547-1 and ‘Horizon’, respectively. Malate levels were categorized subjectively based on the measured malate concentrations [g/L] obtained from fruit bearing genotypes. Each row represents a different parental vine, and columns represent rhAmpSeq markers hierarchically clustered. Cells in colors: Red, yellow and blue, represent different haplotype alleles of a marker, and cells in grey represent missing data.
